# Comparison of fuzzy AHP and fuzzy TODIM methods for landfill location selection

**DOI:** 10.1186/s40064-016-2131-7

**Published:** 2016-04-22

**Authors:** Mohamed Hanine, Omar Boutkhoum, Abdessadek Tikniouine, Tarik Agouti

**Affiliations:** Laboratory of Engineering and Information Systems, Department of Computer Science, Faculty of Sciences Semlalia, Cadi Ayyad University, Marrakesh, Morocco; Team of Telecommunications and Computer Networks, Faculty of Sciences Semlalia, Cadi Ayyad University, Marrakesh, Morocco

**Keywords:** Landfill location selection, Fuzzy logic, Multi-criteria decision-making, Fuzzy AHP, Fuzzy TODIM

## Abstract

Landfill location selection is a multi-criteria decision problem and has a strategic importance for many regions. The conventional methods for landfill location selection are insufficient in dealing with the vague or imprecise nature of linguistic assessment. To resolve this problem, fuzzy multi-criteria decision-making methods are proposed. The aim of this paper is to use fuzzy TODIM (the acronym for Interactive and Multi-criteria Decision Making in Portuguese) and the fuzzy analytic hierarchy process (AHP) methods for the selection of landfill location. The proposed methods have been applied to a landfill location selection problem in the region of Casablanca, Morocco. After determining the criteria affecting the landfill location decisions, fuzzy TODIM and fuzzy AHP methods are applied to the problem and results are presented. The comparisons of these two methods are also discussed.

## Introduction

Landfill location selection of Municipal Solid Waste (MSW) is the determination of a geographic site for a region’s operations concerning waste management. The landfill waste location decision involves governmental authorities seeking to locate or relocate their operations regarding waste management. The process of landfill location decision includes the identification, analysis, evaluation and selection among various alternatives (Liu et al. 2014a). Selecting a location for landfill wastes is a very important decision for each region because it is costly and difficult to reverse, and it involves a long-term commitment. Also location decisions for landfill becomes a challenging task due to many various reasons such as increasing in waste quantities, human population, environmental and public health risk factors, and decreasing in land availability for waste disposal locations (Srivastava and Nema 2012). Hence, decision-makers should select the location that not only has a good performance, but also is flexible enough to accommodate future changes in the regional policy.

Fuzzy set theory integrated with multi-criteria decision making (MCDM) methods has been widely used to deal with uncertainty in the landfill location selection decision process (Beskese et al. 2015), since it provides an appropriate language to manage imprecise criteria, being able to integrate the analysis of qualitative and quantitative factors.

Despite the many researches proposing the use of fuzzy multi criteria decision-making methods, there are no comparative studies of these methods when applied to the problem of landfill location selection. Lima Junior et al. (2014) proposed a comparison of fuzzy AHP and fuzzy TOPSIS methods for supplier selection. In another study, Ertuğrul and Karakaşoğlu (2008) offered a comparison of Fuzzy AHP and Fuzzy TOPSIS methods applied to facility location decision-making. Dehe and Bamford (2015) examined and compared two modelling methods (AHP and ER) for healthcare infrastructure location decision. Ouma et al. (2015) performed a comparison of fuzzy AHP and fuzzy TOPSIS for road pavement maintenance prioritization. However, as the authors point out, there is still a need for a comparative evaluation of MCDM methods in the context of landfill location selection, since the relative advantages of many methods also depend on the characteristics of the problem domain. To overcome this limitation, this paper presents a comparative analysis of the methods fuzzy TODIM and fuzzy AHP applied to the problem of landfill location selection. Comparison of both methods was made on the basis of the analysis of mathematical procedures taking into account the structure of the problem represented by the illustrative application case.

The rest of this paper is organized as follows. The second section, we give a briefly literature review on subject of landfill location selection. The third section, we describe a process of landfill location selection and the main requirements of multi-criteria decision-making methods used in this context. In fourth section , some fundamental concepts regarding fuzzy set theory and the methods fuzzy AHP and fuzzy TODIM are briefly explained. Then in fifth section, a numerical example using both methods in a real case application with the results is presented and the comparative analyses of these results are also illustrated. Finally, conclusions and suggestions for further research are offered in the last section.

## Literature review

Different MCDM approaches have been applied on landfill location selection problem such as AHP, fuzzy AHP, fuzzy ANP, PROMETHEE, fuzzy TOPSIS, OWA, etc., which are used for solving location problems and applied in waste management. Table 1 reviews some major new literature for landfill location selection including authors, dates of publication, approaches used and study area. It is observed that the selection of landfill location is an essential strategic decision, in that it has received more attention in academic literature. A diversity of methodologies and approaches for selecting and evaluating the landfill waste location has been performed (Gbanie et al. 2013; Beskese et al. 2015; Önüt and Soner 2008).

**Table 1:** Major new literature for landfill location selection

Authors	Approach	Study area
Gbanie et al. (2013)	GIS/AHP-OWA-WLC	Bo, Southern Sierra Leone
Khorram et al. (2015)	GIS-fuzzy logic	Bardaskan city, Iran
Khan and Samadder (2015)	GIS/WLC-AHP	Dhanbad, India
Beskese et al. (2015)	Fuzzy AHP and fuzzy TOPSIS	Istanbul, Turkey
El Baba et al. (2015)	GIS-AHP	Gaza Strip, Palestine
Eskandari et al. (2016)	GIS-AHP	Kohgiluyeh and Boyerahmad province, Iran
Rathore et al. (2016)	GIS/SAW-AHP	Lahore District, Pakistan

In real life, the evaluation data of landfill location selection for various subjective criteria and the weights of the criteria are generally expressed in linguistic terms to effectively resolve the ambiguity from available information and do more justice to the essential fuzziness in preference and human judgment. The fuzzy set theory has been used to establish an undefined multiple criteria decision-making problem (Beskese et al. 2015). Thus in current research, fuzzy AHP and fuzzy TODIM methods are proposed for landfill location selection, where the ratings of different alternative locations under different subjective criteria and the weights of all criteria are represented by Triangular Fuzzy Numbers (TFNs).

Multi-criteria decision-making (MCDM) techniques for landfill location selection composed of multi-criteria methods, mathematical programming and stochastic programming (Soltani et al. 2015). There are many different MCDM methods used mostly for evaluation and outranking of alternative locations for landfill waste.

As presented in Table 2, the combination between methods is frequently adopted to deal with the problem of landfill location selection). Fuzzy set theory (Zadeh 1965) has been widely used for modeling decision making processes based on vague and imprecise information such as preferences of decision-makers. The use of optimal methods can bring efficiency and performance to the selection process Ertuğrul and Karakaşoğlu (2008). To determine which methods to use it is necessary to take into account the alignment of the specificities of the problem with the characteristics of the methods (Lima Junior et al. 2014). For example, when selecting a new landfill waste location with many potential locations, methods that do not limit analysis to only a few alternative locations are more suitable than others.

**Table 2:** Decision making approaches applied to landfill location selection

Approach	Method(s)	Proposed by
Single method	AHP	Uyan (2013)
		Tavares et al. (2011)
		El Baba et al. (2015)
	TOPSIS	Yal and Akgün (2013)
	ANP	Afzali et al. (2014)
Combined method	Fuzzy AHP	Donevska et al. (2011)
	Fuzzy AHP and fuzzy TOPSIS	Beskese et al. (2015)
	AHP- WLC	Shahabi et al. (2013)
	AHP-TOPSIS	Demesouka et al. (2013)
	Fuzzy VIKOR	Liu et al. (2014a, b)
	AHP-OWA	Gorsevski et al. (2012)
	Fuzzy ANP	Isalou et al. (2012)
	AHP/fuzzy-TOPSIS	Önüt and Soner (2008)
		Pires et al. (2011)

Other items to take into account to align methods to particularities of landfill location selection are as follows:

- Uncertainty in landfill location selection: the uncertainty in decision making process can refer to the absence of precision of the scores of the relative importance of various criteria as well as the alternative locations. This imprecision may be due to the evaluation by multiple decision-makers in the existence of data on the performance of potential alternatives.
- Flexibility in the decision process: this element concerns mainly the required amount of preferences of the decision-makers in data collection. According to the MCDM method and the number of alternatives and criteria, the quantity of preferences required to collect all the data can make the landfill location selection process very long.
- Adequacy to support decision making group: location selection for landfill decisions are affected by various terms from several functional domains within regional authorities. This assumes that multiple actors from several functional domains participate in the decision making process (Lima Junior et al. 2014). Consequently, it is desirable that the methods used in location selection be optimal to combine several preferences of many decision-makers.
- Computational difficulty: this factor can be linked to either time or space difficulty. The main concern in the landfill waste location selection decision process is connected to time difficulty, which refers to the time in which the algorithm is realized (Chang 1996). Time difficulty varies from method to method as a function of the number of input variables, which in the case of landfill location selection refers to the number of alternative locations and criteria.

Generally, different studies in the literature use the fuzzy AHP method (Donevska et al. 2011) and other fuzzy multi-criteria decision-making (FMCDM) methods (Beskese et al. 2015; Önüt and Soner 2008; Liu et al. 2014b) for landfill location selection. But differently from other studies, fuzzy AHP and fuzzy TODIM methods are proposed for landfill location selection and results are compared in this present contribution.

## Landfill location selection process

**Fig. 1:**
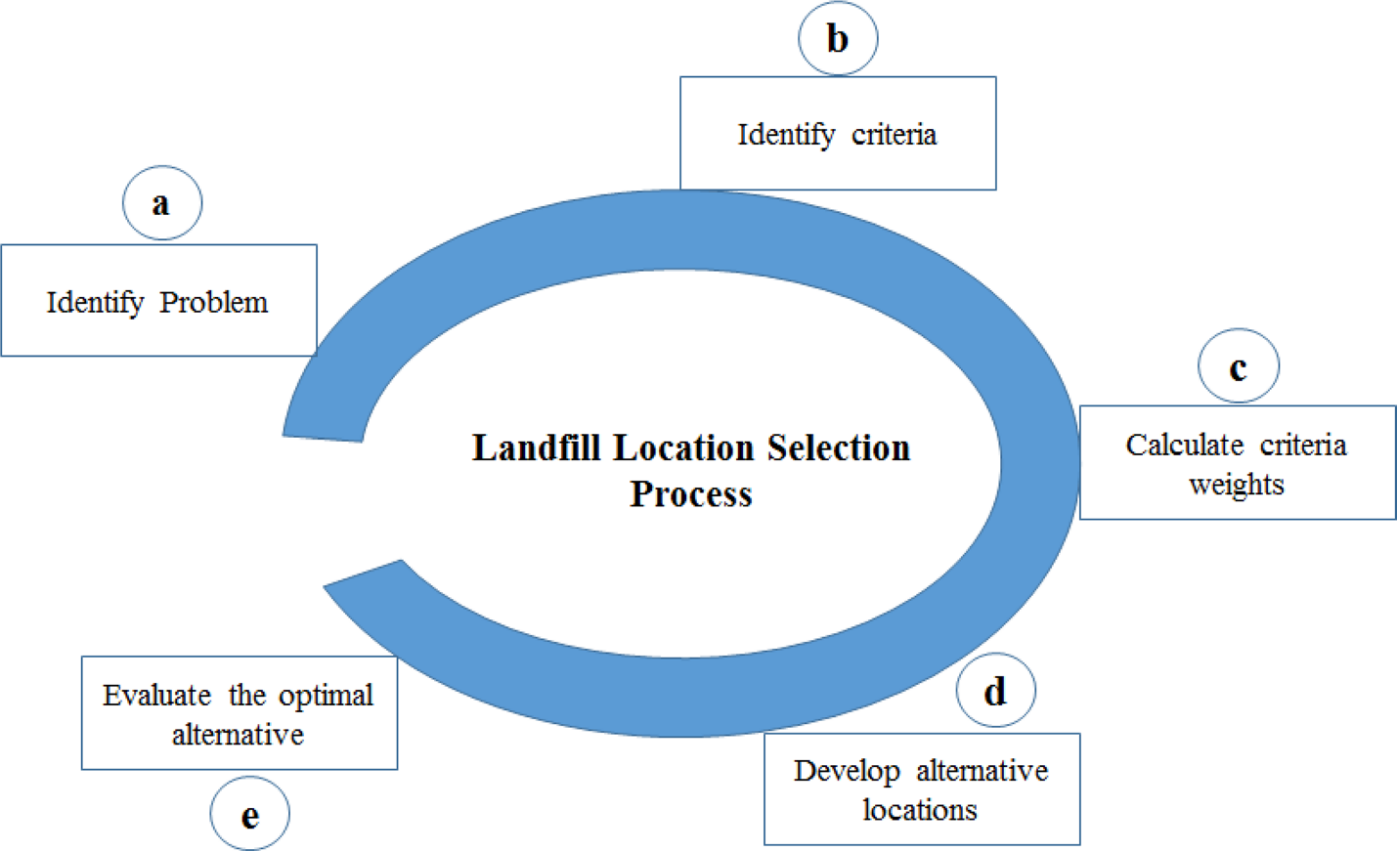
The landfill location selection framework

Landfill location selection is a decision-making process composed of different steps. According to different studies applied by Önüt and Soner (2008), Stevenson (1993), Beskese et al. (2015), Bahrani et al. (2016) and Torabi-Kaveh et al. (2016), which propose many approaches for landfill location selection that consist of five main steps, namely (a) problem definition; (b) identification of criteria that will be used to evaluate and rank location alternatives; (c) calculate criteria weights; (d) develop alternative locations; (e) evaluate the alternative locations and make a decision (Stevenson 1993), as presented in Fig. 1. The objective of the first step is to clarify the problem at hand, which may mean selecting the optimal location for landfill MSW. In this case, selecting new location depending on the number of criteria and alternative locations may be very large. Therefore, this situation requires decision-making techniques that are able to simultaneously evaluate multiple alternatives.

Decision-makers should convert their requirements into decision criteria so as to guide the choices, as the second step of the process. There are several environmental, economic and social–cultural criteria that impact on the location decisions process for landfill which are both quantitative and qualitative. Table 3 indicates some important criteria for landfill location selection. On top of quantitative measures of performance, such as distance from residential areas, Land use or Land cost, and other qualitative measures of evaluation, such as distance from the collect center and access to heavy trucks, are gaining importance (Alves et al. 2009). Therefore, the methods used in the decision process must be able to consider many criteria of both qualitative and quantitative nature (Alves et al. 2009).

**Table 3:** Landfill location performance criteria according to selected authors

	Ground water quality	Available transportation	Soil type	Distance from water bodies	Land cost	Infrastructure cost	Distance from residential areas	Distance from historical areas	Land use	Landscape
Liu et al. (2014a, b)		X			X				X	
Demesouka et al. (2013)	X	X	X	X			X		X	
Alves et al. (2009)	X	X	X	X	X	X	X		X	
Arkoc (2013)	X	X	X						X	
Wang et al. (2009)	X		X	X	X		X			X
Feo and Gisi (2014)	X		X	X			X		X	X
Beskese et al. (2015)	X	X	X	X	X				X	X
Sumathi et al. (2008)	X	X	X	X						X
Şener et al. (2010)	X	X						X		X
Eskandari et al. (2012)	X	X	X	X			X	X		X

In the next step, all weights of the criteria that are selected for landfill location selection are calculated via using a multi-criteria decision-making method. In the fourth step, the main aim is to reduce a set of alternative locations. The last step aims to rank the potential alternatives in order to make the final decision.

## Preliminaries

### Fuzzy set theory

**Fig. 2:**
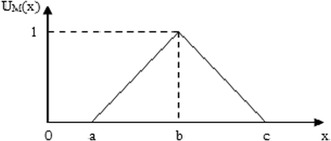
Triangular fuzzy number (TFN)

Fuzzy set theory is among the most preferred theories in decision making, which is an extension of ordinary set theory that was introduced by Zadeh (1965) for dealing with uncertainty and vagueness associated with information. In the literature, trapezoidal and triangular fuzzy numbers that are the forms of fuzzy numbers used in order to capture the vagueness of the parameters related to the topic. In this research work, triangular fuzzy numbers (TFNs) are used. A triangular fuzzy number ẽ (a, b, c) will be used to consider the fuzziness of the landfill waste location selection criteria. The membership function μ(x) of the triangular fuzzy number may therefore be described as Fig. 2 (Beskese et al. 2015; Önüt and Soner 2008).

1 $$\mu_{{\tilde{e}}} ({x}) = \left\{{\begin{array}{*{20}l}{0,}&\quad {{x} \le {a}} \hfill \\ {\frac{{{x} - {a}}}{{{b} - {a}}},} &\quad {{a} < {x} \le {b}} \hfill \\ {\frac{{{c} - {x}}}{{{c} - {b}}},} &\quad {{b} < {x} \le {c}} \hfill \\ {0,}&\quad {{x} > {c}} \hfill \\ \end{array}} \right.$$

The forward of fuzzy set theory used in this study are as follows:

#### **Definition 1**

Let ẽ_1_ (a_1_, b_1_, c_1_) and ẽ_2_ (a_2_, b_2_, c_2_) be two TFNs, then the vertex method is defined to calculate the distance between them as:

2 $${\text{d}}(\tilde{\text{e}}_{1} ,\tilde{\text{e}}_{2} ) = \sqrt {\frac{1}{3}({\text{a}}_{1} - {\text{a}}_{2} )^{2} + ({\text{b}}_{1} - {\text{b}}_{2} )^{2} + ({\text{c}}_{1} - {\text{c}}_{2} )^{2} }$$

#### **Definition 2**

Let ẽ_1_ and ẽ_2_ be two TFNs. The main operations of triangular fuzzy numbers are as follows:

3 $${\tilde{\text{e}}}_{1} \oplus {\tilde{\text{e}}}_{2} = ({\text{a}}_{1} + {\text{a}}_{2} ,{\text{b}}_{1} + {\text{b}}_{2} ,{\text{c}}_{1} + {\text{c}}_{2} )$$

4 $${\tilde{\text{e}}}_{1} - {\tilde{\text{e}}}_{2} = ({\text{a}}_{1} - {\text{a}}_{2} ,{\text{b}}_{1} - {\text{b}}_{2} ,{\text{c}}_{1} - {\text{c}}_{2} )$$

5 $${\tilde{\text{e}}}_{1} \otimes {\tilde{\text{e}}}_{2} = ({\text{a}}_{1} \times {\text{a}}_{2} ,{\text{b}}_{1} \times {\text{b}}_{2} ,{\text{c}}_{1} \times {\text{c}}_{2} )$$

6 $$\frac{{{\tilde{\text{e}}}_{1} }}{{{\tilde{\text{e}}}_{2} }} = \left( {\frac{{{\text{a}}_{1} }}{{{\text{c}}_{2} }},\frac{{{\text{b}}_{1} }}{{{\text{b}}_{2} }},\frac{{{\text{c}}_{1} }}{{{\text{a}}_{2} }}} \right)$$

7 $${\tilde{\text{e}}}_{1} \otimes {\text{k}} = ({\text{a}}_{1} \times {\text{k}},{\text{b}}_{1} \times {\text{k}},{\text{c}}_{1} \times {\text{k}})$$

### Fuzzy AHP

The AHP method developed by Saaty (1980) is widely used for tackling multi-criteria decision problems in real situations. Many works have concluded that AHP is useful and practical for location selection for landfill waste (El Baba et al. 2015; Şener et al. 2010; ZelenovićVasiljević et al. 2011). However, in practice, crisp data are often inadequate to model many situations since human judgments are vague. To overcome classical AHP shortcomings, Van Laarhoven and Pedrycz (1983) proposed fuzzy AHP, which is the combination of analytic hierarchy process (AHP) and Fuzzy Theory. Fuzzy AHP makes it possible to use linguistic ratings in the calculations of criteria weights by giving them a certain range. It is observed that decision-makers are more positive to give interval judgments than fixed-value judgments (Büyüközkan and Ruan 2008). Balli and Korukoglu (2009) recognize that fuzziness in AHP contributes by being able to represent vague and ambiguity information.

There are many procedures for calculating the weights in fuzzy AHP technique proposed in the literature. Brief information about many of these procedures and a concise comparison can be found in (Bozbura et al. 2007). In this study, the extent method introduced by Chang’s (1992) for handling fuzzy AHP, with the use of TFNs is used to calculate the fuzzy weights for the selected criteria (Chang 1996). The outlines of Chang’s extent analysis method on fuzzy AHP have been explained in the following steps (Efe 2016; Boutkhoum et al. 2015):

- **Step 1**: Fuzzy synthetic extent calculation

Let X = {x_1_, x_2_, …, x_n_} be an object set, and G = {g_1_, g_2_, …, g_m_} be a goal set. Using Chang’s extent analysis approach (Chang 1992, 1996), each object is taken on the extent analysis, for each goal, *g*_*i*_, is performed, respectively. Therefore, *m* extent analysis values for each object can be obtained, and are denoted as:

$${\text{M}}_{\text{gi}}^{1},\,\, {\text{M}}_{\text{gi}}^{2}, \ldots, {\text{M}}_{\text{gi}}^{\text{m}}, \quad i = 1,2,3, \ldots, {\text{n}}$$

where all the ${\text{M}}_{\text{gi}}^{\text{m}} (j = 1,2,\ldots,{\text{m}})$ are TFNs.

With respect to the *i*th object, the value of fuzzy synthetic extent is defined as:

8 $${\text{S}}_{\text{i}} = \sum\limits_{{{\text{j}} = 1}}^{\text{m}} {{\text{M}}_{{{\text{g}}_{\text{i}}}}^{\text{j}}} \otimes \left[{\sum\limits_{{{\text{j}} = 1}}^{\text{n}} {\sum\limits_{{{\text{j}} = 1}}^{\text{m}} {{\text{M}}_{\text{gi}}^{\text{j}}}}} \right]^{- 1}$$

To obtain $\sum\nolimits_{{{\text{j}} = 1}}^{\text{m}} {{\text{M}}_{\text{gi}}^{\text{j}} }$ perform the fuzzy addition operation of m extent analysis values for a particular matrix such that

9 $$\sum\limits_{{{\text{j}} = 1}}^{\text{m}} {{\text{M}}_{\text{gi}}^{\text{j}} = \left( {\sum\limits_{{{\text{j}} = 1}}^{\text{m}} {{\text{a}}_{\text{j}} } ,\sum\limits_{{{\text{j}} = 1}}^{\text{m}} {{\text{b}}{}_{\text{j}}} ,\sum\limits_{{{\text{j}} = 1}}^{\text{m}} {{\text{c}}_{\text{j}} } } \right)}$$

And to obtain $\left[ {\sum\nolimits_{{{\text{i}} = 1}}^{\text{n}} {\sum\nolimits_{{{\text{j}} = 1}}^{\text{m}} {{\text{M}}_{\text{gi}}^{\text{j}} } } } \right]{}^{ - 1}$ perform the fuzzy addition operation of values such that ${\text{M}}_{\text{gi}}^{\text{m}} (j = 1,2, \ldots ,{\text{m}})$

10 $$\sum\limits_{{{\text{i}} = 1}}^{\text{n}} {\sum\limits_{{{\text{j}} = 1}}^{\text{m}} {{\text{M}}_{\text{gi}}^{\text{j}}}} = \left({\sum\limits_{{{\text{i}} = 1}}^{\text{n}} {{\text{a}}_{\text{i}}}, \sum\limits_{{{\text{i}} = 1}}^{\text{n}} {{\text{b}}_{\text{i}}}, \sum\limits_{{{\text{i}} = 1}}^{\text{n}} {{\text{c}}_{\text{i}}}} \right)$$

And then compute the inverse of the vector such that

11 $$\left[{\sum\limits_{{{\text{i}} = 1}}^{\text{n}} {\sum\limits_{{{\text{j}} = 1}}^{\text{m}} {{\text{M}}_{{{\text{g}}_{\text{i}}}}^{\text{j}}}}} \right]^{-1} = \left({\frac{1}{{\sum\nolimits_{{{\text{i}} = 1}}^{\text{n}} {{\text{c}}_{\text{i}}}}}, \frac{1}{{\sum\nolimits_{{{\text{i}} = 1}}^{\text{n}} {{\text{b}}_{\text{i}}}}}, \frac{1}{{\sum\nolimits_{{{\text{i}} = 1}}^{\text{n}} {{\text{a}}_{\text{i}}}}}} \right)$$

- **Step 2:** Comparison of fuzzy values

The degree of possibility of M2 = (a_2_, b_2_, c_2_) ≥ M_1_ = (a_1_, b_1_, c_1_) is defined as:

12 $${\text{V}}\left({{\text{M}}_{2} \ge {\text{M}}_{1}} \right) = { \sup }\left[{{ \hbox{min} } \left({\upmu_{{{\text{M}}1}} \left({\text{x}} \right),\upmu_{{{\text{M}}2}} \left({\text{x}} \right)} \right)} \right]$$

**Fig. 3:**
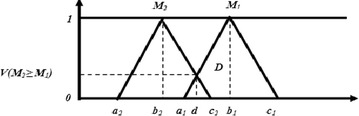
Intersection between M_1_ and M_2_

And can be equivalently expressed as follows:

13 $${\text{V}} ({\text{M2}} \ge {\text{M1}}) ={\text{hgt}} ({\text{M1}} \cap {\text{M2}}) =\left\{\begin{array}{ll} 1, &\quad {\text{if}} \,\, {\text{b}}_{2} > {\text{b}}_{1} \hfill \\0, &\quad {{\text{if}}\;\; {\text{a}}_{1} \ge c_{2}} \\{\frac{{{\text{a}}_{1} - {\text{c}}_{2}}}{{({\text{b}}_{2} -{\text{c}}_{2}) - ({\text{b}}_{1} - {\text{a}}_{1})}},} & \quad {{\text{if}}\;{\text{Otherwise}}} \hfill \\\end{array} \right.$$

where *d* is the ordinate of the highest intersection point *D* between *μ*_*M1*_ and *μ*_*M2*_ as shown in Fig. 3.

For the comparison of M_1_ and M_2_, we need both the values of V(M_1_ ≥ M_2_) and V(M_2_ ≥ M_1_).

- **Step 3:** Priority weight calculation

The degree of possibility for a convex fuzzy number to be greater than *k* convex fuzzy numbers M_i_ (i = 1, 2, 3, …, k) can be defined by:

14 $${\text{V}}\left( {{\text{M}} \ge {\text{M}}_{1} ,{\text{ M}}_{2} , \ldots ,{\text{ M}}_{k} } \right) \, = {\text{ V}}\left[ {\left( {{\text{M }} \ge {\text{ M}}_{1} } \right){\text{ and }}\left( {{\text{M }} \ge {\text{ M}}_{2} } \right){\text{ and }} \ldots \, \left( {{\text{M }} \ge {\text{ M}}_{\text{k}} } \right)} \right]$$

V (M ≥ M_1_, M_2_, …., M_k_) = min V (M ≥ M_i_), if

15 $${\text{m}}\left( {{\text{P}}_{\text{i}} } \right) \, = {\text{ min V }}\left( {{\text{S}}_{\text{i}} \ge {\text{ S}}_{\text{k}} } \right) \, \quad {\text{for}}\;\;{\text{k}} = 1, \, 2, \ldots ,{\text{n}};{\text{ k }} \ne {\text{ i}}.$$

16 $${\text{Then the weight vector is given by:}}\;{\text{ W}}_{\text{p}} = \, \left( {{\text{m}}\left( {{\text{P}}_{1} } \right),{\text{ m}}\left( {{\text{P}}_{2} } \right), \ldots ,{\text{m}}\left( {{\text{P}}_{\text{n}} } \right)} \right)^{\text{T}}$$

where P_i_ (i = 1, 2, …, n) are n elements.

- **Step 4:** Calculation of normalized weight vector
  17 $${\text{W }} = \, \left( {{\text{W}}\left( {{\text{P}}_{1} } \right),{\text{ W}}\left( {{\text{P}}_{2} } \right), \ldots ,{\text{W}}\left( {{\text{P}}_{\text{n}} } \right)} \right)^{\text{T}}$$
  where *W* is a non-fuzzy number.

### Fuzzy TODIM

The TODIM method (an acronym in Portuguese of Interactive and Multi-criteria Decision Making-”Tomada de Decisão Iterativa Multicritério”) is a discrete Multi-Criteria Decision Making (MCDM) method based on Prospect Theory (Kahneman and Tversky 1979) which has awarded the Nobel Prize for Economics in 2002 (Roux 2002). One of the strong characteristics is its capacity to treat risk in MCDM problems.

#### Prospect theory

**Fig. 4:**
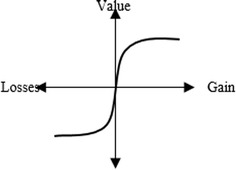
Value function of prospect theory

The value function used in the Prospect Theory is defined in form of a power law depending to the following expression (Kahneman and Tversky 1979; Krohling and de Souza 2012a, b):

18 $${\text{V}} (\chi) = \left\{\begin{array}{*{20}l} {\chi^{\alpha},}&\quad \hbox{if}\,\;\chi \ge 0 \\ {- \theta (- \chi)^{\beta},} &\quad {\hbox{if}\,\; \chi < 0} \hfill \\ \end{array} \right.$$

where α and β are parameters linked to gains and losses, respectively. The parameter θ represents a characteristic of risk factor that is considered in model and must be superior to one. Figure 4 presents a prospect value function with a concave and S-shaped for gains and losses. The values of α = β = 0.88, and θ = 2.25 are experimentally determined by Kahneman and Tversky (1979), which correspond to empirical data. Furthermore, they proposed that the value of θ is between 2.0 and 2.5 (Krohling and de Souza 2012a, b; Gomes et al. 2009).

#### Fuzzy TODIM

To avoid the effects of prejudice of decision-makers and bias in the ranking of alternatives, the fuzzy set theory has been integrated to the traditional TODIM. For expressing the linguistic variables for the attribute values, triangular fuzzy numbers (TFNs) are used. The use of these fuzzy numbers and according to the concept of the TODIM method, gain and loss of each one of the alternatives relative to others are evaluated. Then, by computing the dominance degree of each alternative over the others, the global value of each alternative is obtained and alternatives are ranked. In this paper, the fuzzy TODIM method which was proposed in (Krohling and de Souza 2012a, b; Tosun and Akyüz 2014; Zhang and Fan 2011) is considered. The process steps of this method can be described as follows (Zhang and Xu 2014; Salomon and Rangel 2015):

- **Step 1:** Evaluate the criteria and alternatives

For assessing the criteria and alternatives the linguistic variables are used. To reduce the subjectivity, many decision-makers (experts) should be chosen. After assessments of decision-makers, their scores are integrated. The fuzzy weights of each criterion and fuzzy assessment of each alternative with respect to each criterion can be calculated with the given equations. In the formulas, *k* is the number of decision-makers.

19 $$c{\text{w}}_{\text{j}} = \frac{1}{k}\left[{\sum\limits_{e = 1}^{k} {{\text{cw}}_{\text{j}}^{\text{e}}}} \right]\quad j = 1,2, \ldots,n$$

${\tilde{\text{x}}}_{\text{ij}} =$ The fuzzy evaluation of *i* alternative according to *j* criterion.

20 $$\tilde{x}_{ij} = \frac{1}{k}\left[{\sum\limits_{e = 1}^{k} {\tilde{x}_{ij}^{e}}} \right] \quad i = 1,2, \ldots, m$$

The fuzzy ${\tilde{\text{x}}}_{\text{ij}} =$ values are used as triangular fuzzy numbers in producing the loss and gain matrices.

- **Step 2:** Fuzzy criteria weights (cw_j_) are defuzzificated.

From several techniques of weight normalization, the technique of Abdel-Kader and Dugdale (2001) is used in this paper.

In this technique, the three parameters of triangular fuzzy numbers (a, b, c) for the fuzzy estimates are used. In addition, an index of optimism (α) is used in the classification process. High values in α represents an optimistic decision-maker, while smaller values represent a pessimistic decision-maker.

α parameter is used to present the characteristics of decision-makers, risk taking attitude and various environment conditions. For instance, in a high uncertainty environment a decision-maker with a risk avoiding attitude prefers a lower index of optimism. However, the calculations can be repeated for various values of the index to explore the sensitivity of the decision. In this paper index of optimism (α) is used as 0.5 as a neutral point of view to balance between optimism and pessimism (Tosun and Akyüz 2014).

- Let α ∈ [0, 1] will be index of optimism. For a triangular fuzzy number $\tilde{e}_{j}$ = (a_j_, b_j_, c_j_) (j = 1, 2, …, n);
- Let *V*($\tilde{e}_{j}$) will be the value of $\tilde{e}_{j}$. In this situation, ordering can be calculated as;

21 $$V(\tilde{e}_{j} ) = m_{j} \left[ \begin{aligned} \alpha \left[ {\frac{{c_{j} - x_{{\rm min} } }}{{x_{{\rm max} } - x_{{\rm min} } + c_{j} - b_{j} }}} \right] \hfill \\ + (1 - \alpha )\left[ {1 - \frac{{x_{{\rm max} } - a_{j} }}{{x_{{\rm max} } - x_{{\rm min} } b_{j} - a_{j} }}} \right] \hfill \\ \end{aligned} \right]$$

Here;

22 $$x_{min} = inf \, S$$

23 $$x_{max} = sup \, S$$

24 $$S = U_{j = 1}^{n} S_{j}$$

25 $$S_{l} = \left( {a_{1} ,b_{1} ,c_{1} , \ldots ,a_{n} ,b_{n} ,c_{n} } \right) \quad j = 1,2, \ldots ,n$$

Calculated weights with the classification method are normalized with the given equation:

26 $$w_{j} = \frac{{v(\tilde{e}_{j})}}{{\sum\nolimits_{j = 1}^{n} {v(\tilde{e}_{j})}}}$$

- **Step 3:** Calculate weights (wjr) for each criterion (Cj) based on the reference criterion (Cr)

TODIM technique is based on a projection of the differences between the consequences of any two alternatives to a reference criterion. The criterion with the highest weight value is selected as the reference criterion to translate all pairs of differences between performance measurements into the same dimension. Let C_r_ denote the reference criterion, then the weight w_jr_ of criterion C_j_ to the reference criterion C_r_ may be given as (Fan et al. 2013; Zhang and Fan 2011).

27 $$w_{jr} = w_{j}/w_{r},\quad j \in N \quad {\text{where}} \quad w_{r} = \hbox{max} \left\{{w_{j} j \in N} \right\}$$

- **Step 4:** Determine of Gains and Loses values

To calculate the gain and loss of each alternative according to others, first the values of alternatives should be compared by pair. Let $\tilde{x}_{kj}$ and be $\tilde{x}_{ij}$ the value of alternative *Ai* and *Ak* concerning criterion $C_{j} ,i,k \in M,j \in N$.

Then, gain and loss of alternatives *A*_*i*_ relative to *A*_*k*_ according to criterion *C*_j_ can be given as:

For benefit attribute:

28 $$G_{ik}^{j} = \left\{{\begin{array}{*{20}l} {d(\hat{x}_{ij},\hat{x}_{kj}),} \hfill & {\tilde{x}_{ij} \ge \tilde{x}_{kj},} \hfill \\ {0,} \hfill & {\tilde{x}_{ij} < \tilde{x}_{kj}} \hfill \\ \end{array}} \right\}$$

29 $$L_{ik}^{j} = \left\{{\begin{array}{*{20}l} {0,} \hfill & {\tilde{x}_{ij} \ge \tilde{x}_{kj},} \hfill \\ {- d(\hat{x}_{ij},\hat{x}_{kj}),} \hfill & {\tilde{x}_{ij} < \tilde{x}_{kj}} \hfill \\ \end{array}} \right\}$$

For cost attribute:

30 $$G_{ik}^{j} = \left\{{\begin{array}{*{20}l} {0,} \hfill & \quad {\tilde{x}_{ij} \ge \tilde{x}_{kj},} \hfill \\ {d(\hat{x}_{ij},\hat{x}_{kj}),} \hfill &\quad {\tilde{x}_{ij} < \tilde{x}_{kj}} \hfill \\ \end{array}} \right\}$$

31 $$L_{ik}^{j} = \left\{{\begin{array}{*{20}l} - d(\hat{x}_{ij},\hat{x}_{kj}), & {\tilde{x}_{ij} \ge \tilde{x}_{kj},} \hfill \\ 0, \hfill & {\tilde{x}_{ij} < \tilde{x}_{kj}} \hfill \\ \end{array}} \right\}$$

Using the above equations gain matrix $G_{j} = \left[ {G_{ik}^{j} } \right]_{m \times m}$ and loss matrix $L_{j} = \left[ {L_{ik}^{j} } \right]_{m \times m}$ for criterion *C*j can be elaborated (Fan et al. 2013; Zhang and Fan 2011).

- **Step 5:** Build dominance degree matrix for each criterion (Cj)

First of all, calculate the dominance degree of alternative A_i_ over alternative A_k_ for criterion C_j_. The dominance degree for gain $\Phi _{ik}^{j( + )}$ and dominance degree for loss $\Phi _{ik}^{j( - )}$ can be calculated as follows (Zhang and Xu 2014):

32 $$\Phi _{ik}^{j + } = \sqrt {{{G_{ik}^{j} w_{jr} } \mathord{\left/ {\vphantom {{G_{ik}^{j} w_{jr} } {\left( {\sum\limits_{j = 1}^{n} {w_{jr} } } \right)}}} \right. \kern-0pt} {\left( {\sum\limits_{j = 1}^{n} {w_{jr} } } \right)}},}$$

33 $$\Phi _{ik}^{j( - )} = - \frac{1}{\theta }\sqrt { - L_{ik}^{j} {{\left( {\sum\limits_{j = 1}^{n} {w_{jr} } } \right)} \mathord{\left/ {\vphantom {{\left( {\sum\limits_{j = 1}^{n} {w_{jr} } } \right)} {w_{jr} }}} \right. \kern-0pt} {w_{jr} }},}$$

where θ is the attenuation factor of the loss. Then the dominance degree for the gain and loss ($\Phi _{ik}^{j}$) can be calculated as follows:

34 $$\Phi _{ik}^{j} =\Phi _{ik}^{j( + )} +\Phi _{ik}^{j( - )}$$

By using Eq. (34), dominance degree matrix for criterion *C*_*j*_, $\Phi _{j} = \left[ {\Phi _{ik}^{j} } \right]_{m \times m}$, can be elaborated.

- **Step 6:** Determine overall dominance degree matrix $\left( {\varDelta = \delta ii} \right)$
  35 $$\delta_{ik} = \sum\limits_{j = 1}^{n} {\Phi _{ij}^{j} }$$
- **Step 7:** Calculate overall value of each alternative and rank the alternatives.

Based on matrix Δ, the overall value of alternative can be calculated as follows:

36 $$\delta (A_{i} ) = \frac{{\sum\nolimits_{k = 1}^{m} {\delta_{ik} - \min _{{i \in M}} \left\{ {\sum\nolimits_{k = 1}^{m} {\delta_{ik} } } \right\}} }}{{ \max _{{i \in M}} \left\{ {\sum\nolimits_{k = 1}^{m} {\delta_{ik} } } \right\} - \min _{{i \in M}} \left\{ {\sum\nolimits_{k = 1}^{m} {\delta_{ik} } } \right\}}}$$

Clearly, 0 ≤ (*A*_*i*_) ≤ 1, and the greater (*A*_*i*_) is, the better alternative *A*_*i*_ will be. Consequently, according to descending order of the overall values of all the alternatives, one can determine the ranking of all or chose the desirable alternative(s).

There are only a few applications of TODIM in the literature. For example, Tosun and Akyüz (2014) developed a fuzzy version of the TODIM method for supplier selection problem. Fan et al. (2013) proposed an extended TODIM method to solve the hybrid multiple attribute decision-making (MADM) problems. Ramooshjan et al. (2015) presented a decision-making model for selecting the most appropriate location for the branch of a bank by using a combination of fuzzy set theory and TODIM. De Souza and Krohling (2012) presented a fuzzy TODIM model under group decisions to a relevant problem in crisis management in order to help to select the best combat alternatives based on an accident with oil spill in the sea.

## Application case

The application study is performed to landfill location selection for Casablanca region, which is the most populated and industrial region in Morocco. With its autonomously governed 28 municipalities, Casablanca has a population of 4,270,750. Casablanca has a surface area of 1615 km^2^, which corresponds to 0.23 % of the total surface area of Morocco. The authorities of Casablanca region coordinate solid waste collection, transportation, treatment, and disposal activities. In this region, increasing urbanization and economic development lead to an increase in the quantity of generated solid waste. In fact, there are more than 500 active factories most of which are mostly related to Energy, Pharmaceuticals, Foods, Metal furniture, Plastics and Chemicals and there are relatively few Phosphate derivatives, Oil and Aerospace factories.

**Fig. 5:**
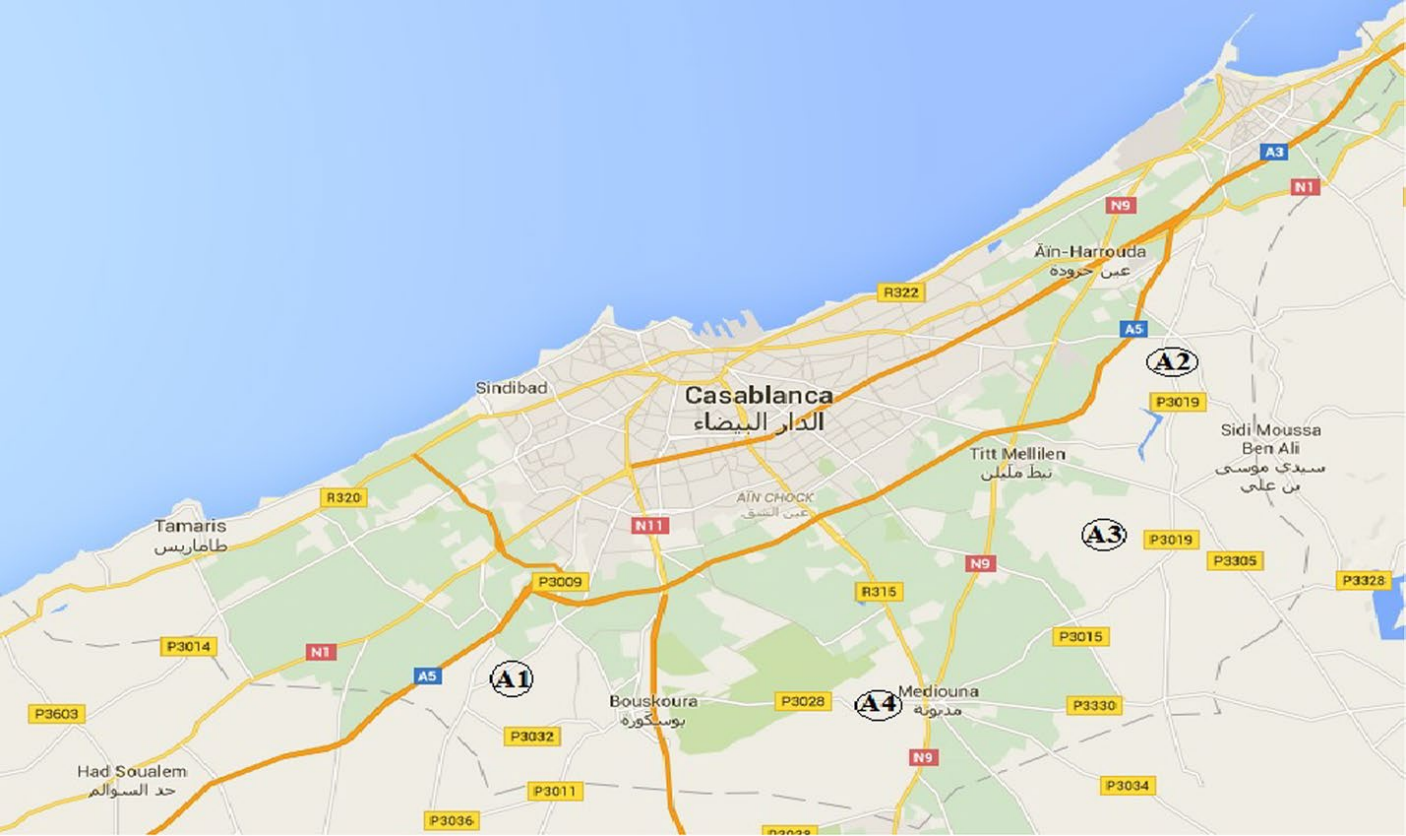
Four feasible location for landfill location selection

**Fig. 6:**
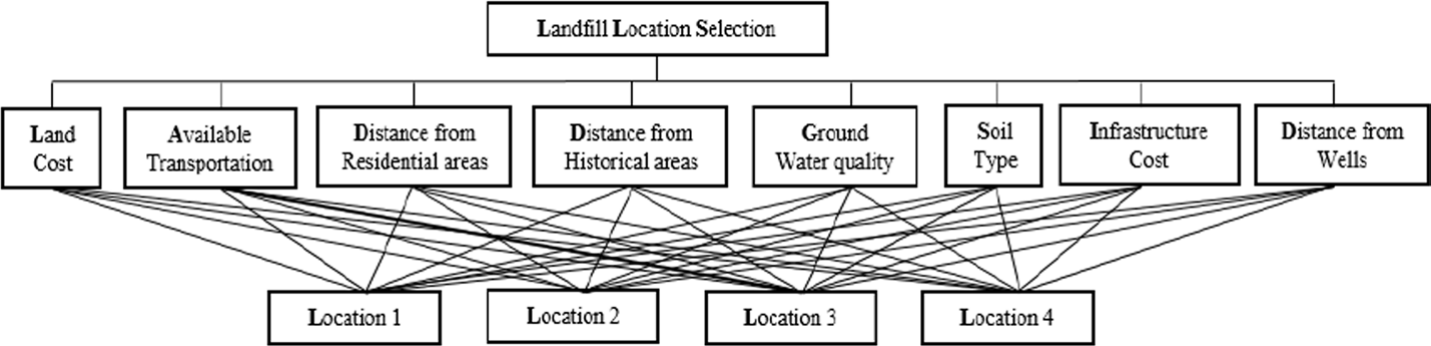
Hierarchical structure for landfill location selection process

On average, the garbage produced in this city is approximately 5000 t daily, which the solid waste of households amounts is approximately 3500 t/day, while the industrial solid waste represents more than 93,000 t/year, and the manufacturing of medical waste around 1030 t/year (Minenv 2013). However, because Casablanca is expected to grow, waste management strategy for the region should be re-evaluated. In the near future, there will be a need for a new landfill location to serve the region. The location of the landfill MSW planned to be constructed would be in or around Casablanca. Firstly, six candidate locations are determined based on GIS information. The landfill location selection commission visits these sites to collect socio-economic and other pertinent information of each site. After these visits, two sites were further eliminated from the list after considering the guidelines of the Moroccan Ministry of Environment. The remaining four feasible sites are as (A_1_, A_2_, A_3_, and A_4_) that are considered for landfill waste (Fig. 5; Vahidi et al. 2013; Tuzkaya et al. 2008). Additionally, a group of decision-makers is consisted. There are three decision-makers (D1, D2, and D3) in this group. Then evaluation criteria are selected with the help of decision-makers’ experiences and literature surveys: land cost (C_1_), available transportation (C_2_), distance from residential areas (C_3_), distance from historical areas (C_4_), ground water quality (C_5_), soil type (C_6_), infrastructure cost (C_7_) and distance from wells (C_8_). The hierarchical structure for the selection of the best alternative location is seen as in Fig. 6.

### Application of Fuzzy TODIM

In this section, fuzzy TODIM method is proposed for the landfill location selection problem in Casablanca region. Firstly, questionnaires are given to the decision-makers for the evaluation process by using the linguistic variables in Table 4. The importance weights of the criteria and the ratings of four alternatives under these criteria determined by these three decision-makers are shown in Tables 5 and 6 respectively.

**Table 4:** Linguistic variables and fuzzy numbers

Linguistic variables	Fuzzy numbers
Very Bad (VB)	(0.00, 0.00, 0.25)
Bad (B)	(0.00, 0.25, 0.50)
Medium (M)	(0.25, 0.50, 0.75)
Good (G)	(0.50, 0.75, 1.00)
Very Good (VG)	(0.75, 1.00, 1.00)

**Table 5:** Importance weight of criteria from three decision-makers

	D1	D2	D3
C1	M	G	VG
C2	B	VG	M
C3	G	B	B
C4	M	M	M
C5	G	G	VG
C6	VB	B	B
C7	VG	G	G
C8	G	M	M

**Table 6:** Ratings of the four alternatives by decision-makers under eight criteria

Criteria	Alternatives	Decision-makers		
		D1	D2	D3
C1	A1	VG	G	VG
	A2	B	G	M
	A3	M	G	VG
	A4	VB	M	B
C2	A1	M	VB	VG
	A2	G	M	G
	A3	VB	B	G
	A4	VB	VG	M
C3	A1	VG	VG	G
	A2	M	B	G
	A3	VG	M	G
	A4	B	VB	M
C4	A1	VB	B	M
	A2	VG	G	G
	A3	B	B	M
	A4	M	VB	VB
C5	A1	VG	VG	G
	A2	G	VG	B
	A3	M	M	G
	A4	G	B	VB
C6	A1	G	VG	VG
	A2	G	M	B
	A3	G	VG	M
	A4	M	B	VB
C7	A1	VG	VB	G
	A2	G	M	G
	A3	B	VB	VB
	A4	VG	G	VG
C8	A1	M	VG	G
	A2	B	M	VB
	A3	M	B	G
	A4	VG	VG	M

Then linguistic variables shown in Tables 5 and 6 are transformed into triangular fuzzy numbers (TFNs) to form fuzzy decision matrix as shown in Table 7.

**Table 7:** Aggregated fuzzy weights of the criteria and alternatives

	A1	A2	A3	A4	Fuzzy weight criteria w_j_
C1	(0.66;0.92;1)	(0.25;0.5;0.75)	(0.5;0.75;0.92)	(0.08;0.25;0.5)	(0.5;0.75;0.91)
C2	(0.33;0.5;0.66)	(0.41;0.66;0.92)	(0.33;0.58;0.83)	(0.33;0.5;0.66)	(0.33;0.58;0.75)
C3	(0.66;0.92;1)	(0.25;0.5;0.75)	(0.5;0.75;0.92)	(0.08;0.25;0.5)	(0.16;0.75;0.66)
C4	(0.08;0.25;0.5)	(0.58;0.83;1)	(0.08;0.33;0.58)	(0.08;0.16;0.41)	(0.25;0.5;0.75)
C5	(0.66;0.92;1)	(0.41;0.66;0.83)	(0.33;0.58;0.83)	(0.16;0.33;0.58)	(0.58;0.83;1)
C6	(0.66;0.92;1)	(0.25;0.5;0.75)	(0.5;0.75;0.92)	(0.08;0.25;0.5)	(0;0.16;0.14)
C7	(0.41;0.58;0.75)	(0.41;0.66;0.92)	(0;0.083;0.33)	(0.66;0.92;1)	(0.58;1.16;1)
C8	(0.5;0.75;0.92)	(0.08;0.25;0.5)	(0.25;0.5;0.75)	(0.58;0.83;0.91)	(0.33;0.58;0.83)

Weights of criteria (w_j_) given in Table 6 are defuzzicated by using Eqs. (21)–(26) and normalized. Table 8 presents relative weights of criteria (w_jr_) that are determined by Eq. (27), in which C_7_ is chosen as the reference criterion (Tosun and Akyüz 2014).

**Table 8:** Relative weights of the criteria

Criteria	*W*_*j*_	*W*_*jr*_
C1	0.144	0.475
C2	0.089	0.294
C3	0.123	0.406
C4	0.07	0.231
C5	0.175	0.577
C6	0.006	0.020
C7	0.303	1
C8	0.091	0.300

The next step is dedicated to calculate the gain and loss matrices for each criterion by using the fuzzy values in Table 7 and with Eqs. (28)–(31).

$${\text{G1}} = \left| {\begin{array}{*{20}l} 0 \hfill & \quad {0.368} \hfill & \quad {0.142} \hfill & \quad {0.587} \hfill \\ 0 \hfill & \quad 0 \hfill & \quad 0 \hfill & \quad {0.226} \hfill \\ 0 \hfill & \quad {0.226} \hfill & \quad 0 \hfill & \quad {0.448} \hfill \\ 0 \hfill & \quad 0 \hfill & \quad 0 \hfill & \quad 0 \hfill \\ \end{array} } \right|$$

$${\text{L1}} = \left| {\begin{array}{*{20}l} 0 \hfill & \quad 0 \hfill & \quad 0 \hfill & \quad 0 \hfill \\ { - 0.368} \hfill & \quad 0 \hfill & \quad { - 0.226} \hfill & \quad 0 \hfill \\ { - 0.142} \hfill & \quad 0 \hfill & \quad 0 \hfill & \quad 0 \hfill \\ { - 0.587} \hfill & \quad { - 0.226} \hfill & \quad { - 0.448} \hfill & \quad 0 \hfill \\ \end{array} } \right|$$

$${\text{G2}} = \left| {\begin{array}{*{20}l} 0 \hfill & \quad 0 \hfill & \quad 0 \hfill & \quad 0 \hfill \\ {0.182} \hfill & \quad 0 \hfill & \quad {0.083} \hfill & \quad {0.182} \hfill \\ {0.108} \hfill & \quad 0 \hfill & \quad 0 \hfill & \quad {0.108} \hfill \\ 0 \hfill & \quad 0 \hfill & \quad 0 \hfill & \quad 0 \hfill \\ \end{array} } \right|$$

$${\text{L2}} = \left| {\begin{array}{*{20}l} 0 \hfill & \quad { - 0.182} \hfill & \quad { - 0.108} \hfill & \quad 0 \hfill \\ 0 \hfill & \quad 0 \hfill & \quad 0 \hfill & \quad { - 0.083} \hfill \\ 0 \hfill & \quad { - 0.083} \hfill & \quad 0 \hfill & \quad 0 \hfill \\ 0 \hfill & \quad { - 0.182} \hfill & \quad { - 0.108} \hfill & \quad 0 \hfill \\ \end{array} } \right|$$

$${\text{G3}} = \left| {\begin{array}{*{20}l} 0 \hfill & \quad {0.368} \hfill & \quad {0.142} \hfill & \quad {0.587} \hfill \\ 0 \hfill & \quad 0 \hfill & \quad 0 \hfill & \quad {0.226} \hfill \\ 0 \hfill & \quad {0.226} \hfill & \quad 0 \hfill & \quad {0.448} \hfill \\ 0 \hfill & \quad 0 \hfill & \quad 0 \hfill & \quad 0 \hfill \\ \end{array} } \right|$$

$${\text{L3}} = \left| {\begin{array}{*{20}l} 0 \hfill & \quad 0 \hfill & \quad 0 \hfill & \quad 0 \hfill \\ { - 0.368} \hfill & \quad 0 \hfill & \quad { - 0.226} \hfill & \quad 0 \hfill \\ { - 0.142} \hfill & \quad 0 \hfill & \quad 0 \hfill & \quad 0 \hfill \\ { - 0.587} \hfill & \quad { - 0.226} \hfill & \quad { - 0.448} \hfill & \quad 0 \hfill \\ \end{array} } \right|$$

$${\text{G4}} = \left| {\begin{array}{*{20}l} 0 \hfill & \quad 0 \hfill & \quad 0 \hfill & \quad {0.073} \hfill \\ {0.528} \hfill & \quad 0 \hfill & \quad {0.474} \hfill & \quad {0.590} \hfill \\ {0.065} \hfill & \quad 0 \hfill & \quad 0 \hfill & \quad {0.138} \hfill \\ 0 \hfill & \quad 0 \hfill & \quad 0 \hfill & \quad 0 \hfill \\ \end{array} } \right|$$

$${\text{L4}} = \left| {\begin{array}{*{20}l} 0 \hfill & \quad { - 0.528} \hfill & \quad {0.065} \hfill & \quad 0 \hfill \\ 0 \hfill & \quad 0 \hfill & \quad 0 \hfill & \quad { - 0.474} \hfill \\ 0 \hfill & \quad { - 0.474} \hfill & \quad 0 \hfill & \quad 0 \hfill \\ { - 0.073} \hfill & \quad { - 0.590} \hfill & \quad { - 0.138} \hfill & \quad 0 \hfill \\ \end{array} } \right|$$

$${\text{G5}} = \left| {\begin{array}{*{20}l} 0 \hfill & \quad {0.230} \hfill & \quad {0.290} \hfill & \quad {0.508} \hfill \\ 0 \hfill & \quad 0 \hfill & \quad {0.065} \hfill & \quad {0.279} \hfill \\ 0 \hfill & \quad 0 \hfill & \quad 0 \hfill & \quad {0.226} \hfill \\ 0 \hfill & \quad 0 \hfill & \quad 0 \hfill & \quad 0 \hfill \\ \end{array} } \right|$$

$${\text{L5}} = \left| {\begin{array}{*{20}l} 0 \hfill & \quad 0 \hfill & \quad 0 \hfill & \quad 0 \hfill \\ { - 0.230} \hfill & \quad 0 \hfill & \quad 0 \hfill & \quad { - 0.065} \hfill \\ { - 0.290} \hfill & \quad { - 0.065} \hfill & \quad 0 \hfill & \quad 0 \hfill \\ { - 0.508} \hfill & \quad { - 0.279} \hfill & \quad { - 0.226} \hfill & \quad 0 \hfill \\ \end{array} } \right|$$

$${\text{G6}} = \left| {\begin{array}{*{20}l} 0 \hfill & \quad {0.368} \hfill & \quad {0.142} \hfill & \quad {0.587} \hfill \\ 0 \hfill & \quad 0 \hfill & \quad 0 \hfill & \quad {0.226} \hfill \\ 0 \hfill & \quad {0.226} \hfill & \quad 0 \hfill & \quad {0.448} \hfill \\ 0 \hfill & \quad 0 \hfill & \quad 0 \hfill & \quad 0 \hfill \\ \end{array} } \right|$$

$${\text{L6}} = \left| {\begin{array}{*{20}l} 0 \hfill & \quad 0 \hfill & \quad 0 \hfill & \quad 0 \hfill \\ { - 0.368} \hfill & \quad 0 \hfill & \quad { - 0.226} \hfill & \quad 0 \hfill \\ { - 0.142} \hfill & \quad 0 \hfill & \quad 0 \hfill & \quad 0 \hfill \\ { - 0.587} \hfill & \quad { - 0.226} \hfill & \quad { - 0.448} \hfill & \quad 0 \hfill \\ \end{array} } \right|$$

$${\text{G7}} = \left| {\begin{array}{*{20}l} 0 \hfill & \quad 0 \hfill & \quad {0.444} \hfill & \quad 0 \hfill \\ {0.108} \hfill & \quad 0 \hfill & \quad {0.532} \hfill & \quad 0 \hfill \\ 0 \hfill & \quad 0 \hfill & \quad 0 \hfill & \quad 0 \hfill \\ {0.283} \hfill & \quad {0.213} \hfill & \quad {0.726} \hfill & \quad 0 \hfill \\ \end{array} } \right|$$

$${\text{L7}} = \left| {\begin{array}{*{20}l} 0 \hfill & \quad { - 0.108} \hfill & \quad 0 \hfill & \quad { - 0.283} \hfill \\ 0 \hfill & \quad 0 \hfill & \quad 0 \hfill & \quad { - 0.532} \hfill \\ { - 0.444} \hfill & \quad { - 0.532} \hfill & \quad 0 \hfill & \quad { - 0.726} \hfill \\ 0 \hfill & \quad 0 \hfill & \quad 0 \hfill & \quad 0 \hfill \\ \end{array} } \right|$$

$${\text{G8}} = \left| {\begin{array}{*{20}l} 0 \hfill & \quad {0.448} \hfill & \quad {0.226} \hfill & \quad 0 \hfill \\ 0 \hfill & \quad 0 \hfill & \quad 0 \hfill & \quad 0 \hfill \\ 0 \hfill & \quad {0.226} \hfill & \quad 0 \hfill & \quad 0 \hfill \\ 0 \hfill & \quad {0.501} \hfill & \quad {0.284} \hfill & \quad 0 \hfill \\ \end{array} } \right|$$

$${\text{L8}} = \left| {\begin{array}{*{20}l} 0 \hfill & \quad 0 \hfill & \quad 0 \hfill & \quad { - 0.065} \hfill \\ { - 0.448} \hfill & \quad 0 \hfill & \quad { - 0.226} \hfill & \quad 0 \hfill \\ { - 0.226} \hfill & \quad 0 \hfill & \quad 0 \hfill & \quad { - 0.284} \hfill \\ { - 0.065} \hfill & \quad 0 \hfill & \quad 0 \hfill & \quad 0 \hfill \\ \end{array} } \right|$$

Dominance degree matrices for each attributes are calculated as follow by using the Eqs. (32)–(34):

$$\Phi_{1} = \left| {\begin{array}{*{20}l} 0 \hfill & \quad {0.230} \hfill & \quad {0.143} \hfill & \quad {0.290} \hfill \\ { - 0.23} \hfill & \quad 0 \hfill & \quad { - 0.180} \hfill & \quad {0.18} \hfill \\ { - 0.143} \hfill & \quad {0.18} \hfill & \quad 0 \hfill & \quad {0.253} \hfill \\ { - 0.29} \hfill & \quad { - 0.18} \hfill & \quad { - 0.253} \hfill & \quad 0 \hfill \\ \end{array} } \right|$$

$$\Phi_{2} = \left| {\begin{array}{*{20}l} 0 \hfill & \quad { - 0.127} \hfill & \quad { - 0.098} \hfill & \quad 0 \hfill \\ {0.127} \hfill & \quad 0 \hfill & \quad {0.086} \hfill & \quad {0.041} \hfill \\ {0.098} \hfill & \quad { - 0.086} \hfill & \quad 0 \hfill & \quad {0.098} \hfill \\ 0 \hfill & \quad { - 0.127} \hfill & \quad { - 0.098} \hfill & \quad 0 \hfill \\ \end{array} } \right|$$

$$\Phi_{3} = \left| {\begin{array}{*{20}l} 0 \hfill & \quad {0.212} \hfill & \quad {0.132} \hfill & \quad {0.268} \hfill \\ { - 0.212} \hfill & \quad 0 \hfill & \quad { - 0.166} \hfill & \quad {0.166} \hfill \\ { - 0.132} \hfill & \quad {0.166} \hfill & \quad 0 \hfill & \quad {0.234} \hfill \\ { - 0.268} \hfill & \quad { - 0.166} \hfill & \quad { - 0.234} \hfill & \quad 0 \hfill \\ \end{array} } \right|$$

$$\Phi_{4} = \left| {\begin{array}{*{20}l} 0 \hfill & \quad { - 0.192} \hfill & \quad { - 0.067} \hfill & \quad {0.071} \hfill \\ {0.192} \hfill & \quad 0 \hfill & \quad {0.182} \hfill & \quad {0.021} \hfill \\ {0.067} \hfill & \quad { - 0.182} \hfill & \quad 0 \hfill & \quad {0.098} \hfill \\ { - 0.071} \hfill & \quad { - 0.203} \hfill & \quad { - 0.098} \hfill & \quad 0 \hfill \\ \end{array} } \right|$$

$$\Phi_{5} = \left| {\begin{array}{*{20}l} 0 \hfill & \quad {0.200} \hfill & \quad {0.225} \hfill & \quad {0.297} \hfill \\ { - 0.200} \hfill & \quad 0 \hfill & \quad {0.106} \hfill & \quad {0.114} \hfill \\ { - 0.225} \hfill & \quad { - 0.106} \hfill & \quad 0 \hfill & \quad {0.198} \hfill \\ { - 0.297} \hfill & \quad { - 0.220} \hfill & \quad { - 0.198} \hfill & \quad 0 \hfill \\ \end{array} } \right|$$

$$\Phi_{6} = \left| {\begin{array}{*{20}l} 0 \hfill & \quad {0.047} \hfill & \quad {0.029} \hfill & \quad {0.059} \hfill \\ { - 0.047} \hfill & \quad 0 \hfill & \quad { - 0.037} \hfill & \quad {0.037} \hfill \\ { - 0.029} \hfill & \quad {0.037} \hfill & \quad 0 \hfill & \quad {0.052} \hfill \\ { - 0.059} \hfill & \quad { - 0.037} \hfill & \quad { - 0.052} \hfill & \quad 0 \hfill \\ \end{array} } \right|$$

$$\Phi_{7} = \left| {\begin{array}{*{20}l} 0 \hfill & \quad { - 0.181} \hfill & \quad {0.366} \hfill & \quad { - 0.292} \hfill \\ {0.181} \hfill & \quad 0 \hfill & \quad {0.401} \hfill & \quad { - 0.401} \hfill \\ { - 0.366} \hfill & \quad { - 0.401} \hfill & \quad 0 \hfill & \quad { - 0.469} \hfill \\ {0.292} \hfill & \quad {0.254} \hfill & \quad {0.469} \hfill & \quad 0 \hfill \\ \end{array} } \right|$$

$$\Phi_{8} = \left| {\begin{array}{*{20}l} 0 \hfill & \quad {0.201} \hfill & \quad {0.143} \hfill & \quad { - 0.077} \hfill \\ { - 0.201} \hfill & \quad 0 \hfill & \quad { - 0.143} \hfill & \quad 0 \hfill \\ { - 0.143} \hfill & \quad {0.143} \hfill & \quad 0 \hfill & \quad { - 0.160} \hfill \\ { - 0.077} \hfill & \quad {0.213} \hfill & \quad {0.160} \hfill & \quad 0 \hfill \\ \end{array} } \right|$$

In this paper, the value of θ is given 1, which means that losses will contribute with their real values to the overall value. Using Eq. (35), global dominance matrix can be found:

$$\Delta = \left| {\begin{array}{*{20}l} 0 \hfill & \quad {0.391} \hfill & \quad {0.874} \hfill & \quad {0.618} \hfill \\ { - 0.3917} \hfill & \quad 0 \hfill & \quad {0.248} \hfill & \quad {0.159} \hfill \\ { - 0.874} \hfill & \quad { - 0.248} \hfill & \quad 0 \hfill & \quad {0.306} \hfill \\ { - 0.772} \hfill & \quad { - 0.468} \hfill & \quad { - 0.306} \hfill & \quad 0 \hfill \\ \end{array} } \right|$$

Finally, by using the Eq. (36) the global value of each alternative can be obtained as (*A*_1_) = 1, (*A*_2_) = 0.4556, (*A*_3_) = 0.2129, and (*A*_4_) = 0. With respect to these values of four alternatives, the ranking is determined as A_1_ > A_2_ > A_3_ > A_4_.

### Application with fuzzy AHP method

In this section, fuzzy AHP method is proposed for the same problem of the landfill location selection in Casablanca region. We proposed a group of decision-makers based on fuzzy AHP. The linguistic terms and TFN values used to comparatively evaluate the weight of the criteria and the ratings of the alternatives are presented in Table 9.

**Table 9:** Triangular Fuzzy Numbers of linguistic comparison

Linguistics terms	Triangular fuzzy numbers (TFN)
Extremely more importance(EMI)	(8, 9, 10)
Very strong importance (VSI)	(6, 7, 8)
Strong importance (SI)	(4, 5, 6)
Moderate importance (MI)	(2, 3, 4)
Equal importance (EI)	(1, 1, 2)

Table 10 shows the comparison judgments of the weights of the criteria made by the three decision-makers involved already are transformed into TFN. The results of aggregation of these fuzzy values are shown in Table 11 and were obtained by the geometric mean of the judgments by using the Eq. (37) (Chang et al. 2009; Büyüközkan and Feyzıog̃lu 2004):

37 $$a_{ij} = \hbox{min} \left( {a_{ijk} } \right),\quad b_{ij} = \left( {\prod\limits_{k = 1}^{k} {b_{ijk} } } \right)^{1/k} ,\quad c_{ij} = \hbox{max} \left( {c_{ijk} } \right)$$

where (a_ijy_, b_ijy_, c_ijy_) is the fuzzy evaluation of sample members k (k = 1, 2, …, k).

**Table 10:** Comparative judgments of the weights of the criteria realized by decision makers

		C1	C2	C3	C4	C5	C6	C7	C8
C1	D1	(1,1,1)	(2,3,4)	(4,5,6)	(8,9,10)	(0.25,0.33,0.5)	(6,7,8)	(0.17,0.2,0.25)	(2,3,4)
	D2	(1,1,1)	(1,1,2)	(2,3,4)	(2,3,4)	(0.12,0.14,0.17)	(6,7,8)	(0.17,0.2,0.25)	(4,5,6)
	D3	(1,1,1)	(4,5,6)	(0.17,0.2,0.25)	(2,3,4)	(4,5,6)	(6,7,8)	(1,1,2)	(0.13,0.15,0.17)
C2	D1	(0.25;0.33;0.5)	(1,1,1)	(4,5,6)	(6,7,8)	(1,1,2)	(0.25,0.33,0.5)	(2,3,4)	(2,3,4)
	D2	(1,1,2)	(1,1,1)	(0.25,0.33,0.5)	(8,9,10)	(4,5,6)	(2,3,4)	(2,3,4)	(0.25,0.33,0.5)
	D3	(0.17;0.2;0.25)	(1,1,1)	(0.25,0.33,0.5)	(2,3,4)	(8,9,10)	(2,3,4)	(0.17,0.2,0.25)	(4,5,6)
C3	D1	(0.17;0.2;0.25)	(0.17,0.2,0.25)	(1,1,1)	(8,9,10)	(1,1,2)	(4,5,6)	(0.17,0.2,0.25)	(4,5,6)
	D2	(0.25,0.33,0.5)	(2,3,4)	(1,1,1)	(0.12,0.4,0.17)	(0.12,0.4,0.17)	(0.25,0.33,0.5)	(4,5,6)	(0.1,0.11,0.13)
	D3	(4,5,6)	(2,3,4)	(1,1,1)	(8,9,10)	(0.17,0.2,0.25)	(2,3,4)	(0.17,0.2,0.25)	(0.12,0.14,0.17)
C4	D1	(0.4,0.11,0.13)	(0.12,0.14,0.17)	(0.1,0.11,0.13)	(1,1,1)	(0.17,0.2,0.25)	(4,5,6)	(4,5,6)	(2,3,4)
	D2	(0.25,0.33,0.5)	(0.1,0.11,0.13)	(6,7,8)	(1,1,1)	(6,7,8)	(0.17,0.2,0.25)	(1,1,2)	(0.12,0.14,0.17)
	D3	(0.25,0.33,0.5)	(0.25,0.33,0.5)	(0.1,0.11,0.13)	(1,1,1)	(1,1,2)	(8,9,10)	(0.25,0.33,0.5)	(1,1,2)
C5	D1	(2,3,4)	(1,1,2)	(1,1,2)	(4,5,6)	(1,1,1)	(1,1,2)	(4,5,6)	(2,3,4)
	D2	(6,7,8)	(0.17,0.2,0.25)	(6,7,8)	(0.12,0.14,0.17)	(1,1,1)	(0.25,0.33,0.5)	(2,3,4)	(8,9,10)
	D3	(0.17,0.2,0.25)	(0.1,0.11,0.13)	(4,5,6)	(1,1,2)	(1,1,1)	(4,5,6)	(2,3,4)	(0.12,0.14,0.17)
C6	D1	(0.12,0.14,0.17)	(2,3,4)	(0.17,0.2,0.25)	(0.17,0.2,0.25)	(1,1,2)	(1,1,1)	(0.1,0.11,0.13)	(2,3,4)
	D2	(0.12,0.14,0.17)	(0.25,0.33,0.5)	(2,3,4)	(4,5,6)	(2,3,4)	(1,1,1)	(0.17,0.2,0.25)	(6,7,8)
	D3	(0.12,0.14,0.17)	(0.25,0.33,0.5)	(0.25,0.33,0.5)	(0.1,0.11,0.13)	(0.17,0.2,0.25)	(1,1,1)	(1,1,2)	(6,7,8)
C7	D1	(4,5,6)	(0.25,0.33,0.5)	(4,5,6)	(0.17,0.2,0.25)	(0.17,0.2,0.25)	(8,9,10)	(1,1,1)	(0.25,0.33,0.5)
	D2	(4,5,6)	(0.25,0.33,0.5)	(0.17,0.2,0.25)	(1,1,2)	(0.25,0.33,0.5)	(4,5,6)	(1,1,1)	(0.1,0.11,0.13)
	D3	(1,1,2)	(4,5,6)	(4,5,6)	(2,3,4)	(0.25,0.33,0.5)	(1,1,2)	(1,1,1)	(8,9,10)
C8	D1	(0.25,0.33,0.5)	(0.25,0.33,0.5)	(0.17,0.2,0.25)	(0.25,0.33,0.5)	(0.25,0.33,0.5)	(0.25,0.33,0.5)	(2,3,4)	(1,1,1)
	D2	(0.17,0.2,0.25)	(2,3,4)	(8,9,10)	(6,7,8)	(0.1,0.11,0.13)	(0.12,0.14,0.17)	(8,9,10)	(1,1,1)
	D3	(6,7,8)	(0.17,0.2,0.25)	(6,7,8)	(1,1,2)	(6,7,8)	(0.12,0.14,0.17)	(0.1,0.11,0.13)	(1,1,1)

**Table 11:** Fuzzy numbers of the aggregated weights of the criteria

	C1	C2	C3	C4	C5	C6	C7	C8
C1	(1,1,1)	(1,2.46,6)	(0.16,1.44,6)	(2,4.32,10)	(0.12,0.62,6)	(6,7,8)	(0.17,0.34,2)	(0.12,1.28,6)
C2	(0.17,0.4,2)	(1,1,1)	(0.25,0.86,6)	(2,5.74,10)	(1,3.55,10)	(0.25,1.44,4)	(0.17,1.21,4)	(0.25,1.71,6)
C3	(0.17,0.7,6)	(0.17,1.21,4)	(1,1,1)	(0.12,2.26,10)	(0.12,0.31,2)	(0.25,1.11,6)	(0.17,0.59.6)	(0.1,0.42,6)
C4	(0.1,0.23,0.5)	(0.1,0.17,0.5)	(0.1,0.44,8)	(1,1,1)	(0.17,1.2,8)	(0.17,2.1,10)	(0.25,1.19,6)	(0.12,0.75,4)
C5	(0.17,1.61,8)	(0.1,0.28,2)	(1,3.27,8)	(0.12,0.9,6)	(1,1,1)	(0.25,1.19,6)	(2,3.55,6)	(0.12,1.56,10)
C6	(0.12,0.14,0.17)	(0.25,0.7,4)	(0.17,0.6,4)	(0.1,0.5,6)	(0.17,0.84,4)	(1,1,1)	(0.1,0.28,2)	(2,5.28,8)
C7	(1,2.92,6)	(0.25,0.82,6)	(0.17,1.71,6)	(0.17,0.4,4)	(0.17,0.28,0.5)	(1,3.56,10)	(1,1,1)	(0.1,0.69,10)
C8	(0.17,0.77,8)	(0.17,0.58,4)	(0.17,2.32,10)	(0.25,1.33,8)	(0.1,0.64,8)	(0.12,0.19,0.5)	(0.1,1.44,10)	(1,1,1)

Similarly, the fuzzy values of the aggregated comparative judgments of the alternative locations according to each criterion made by the three decision-makers are shown in Tables 12, 13, 14, 15, 16, 17, 18 and 19.

**Table 12:** Fuzzy numbers of the alternative locations ratings related to criterion C1

	A1	A2	A3	A4
A1	(1,1,1)	(0.25,2.26,8)	(1,1,2)	(0.25,1.91,8)
A2	(0.12,0.44,4)	(1,1,1)	(0.16,1.75,10)	(0.16,0.58,4)
A3	(1,1,2)	(0.10,0.57,6)	(1,1,1)	(0.16,1.91,8)
A4	(0.12,0.52,4)	(0.25,1.70,6)	(0.12,0.52,6)	(1,1,1)

**Table 13:** Fuzzy numbers of the alternative locations ratings related to criterion C2

	A1	A2	A3	A4
A1	(1,1,1)	(0.25,2.26,8)	(0.25,1.70,6)	(2,5.13,10)
A2	(0.12,0.44,4)	(1,1,1)	(0.25,1.70,6)	(0.25,2.53,8)
A3	(0.16,0.58,4)	(0.16,0.58,4)	(1,1,1)	(0.16,0.58,2)
A4	(0.10,0.19,0.50)	(0.12,0.39,4)	(1,1.71,6)	(1,1,1)

**Table 14:** Fuzzy numbers of the alternative locations ratings related to criterion C3

	A1	A2	A3	A4
A1	(1,1,1)	(4,5.59,8)	(0.25,1,4)	(2,5.27,8)
A2	(0.12,0.17,0.25)	(1,1,1)	(1,3.27,8)	(1,1.91,8)
A3	(0.25,1,4)	(0.12,0.30,2)	(1,1,1)	(0.25,1.18,6)
A4	(0.12,0.18,0.50)	(0.12,0.52,2)	(0.16,0.84,4)	(1,1,1)

**Table 15:** Fuzzy numbers of the alternative locations ratings related to criterion C4

	A1	A2	A3	A4
A1	(1,1,1)	(4,5.59,8)	(1,1.44,4)	(4,5.59,8)
A2	(0.12,0.17,0.25)	(1,1,1)	(4,5,6)	(0.25,2.46,10)
A3	(0.25,0.69,2)	(0.16,0.2,0.25)	(1,1,1)	(1,2.08,4)
A4	(0.12,0.17,0.25)	(0.1,0.4,0.2)	(0.25,0.48,2)	(1,1,1)

**Table 16:** Fuzzy numbers of the alternative locations ratings related to criterion C5

	A1	A2	A3	A4
A1	(1,1,1)	(1,3.65,8)	(0.25,1.18,6)	(0.25,1.19,8)
A2	(0.12,0.27,2)	(1,1,1)	(1,1.71,6)	(0.16,0.69,6)
A3	(0.16,0.84,4)	(0.16,0.58,2)	(1,1,1)	(0.12,0.89,6)
A4	(0.12,0.52,4)	(0.16,1.44,6)	(0.16,1.11,8)	(1,1,1)

**Table 17:** Fuzzy numbers of the alternative locations ratings related to criterion C6

		A1	A2	A3	A4
A1		(1,1,1)	(0.25,1,4)	(0.12,0.75,10)	(0.16,2.14,8)
A2		(0.25,1,4)	(1,1,1)	(1,2.75,8)	(1,1.71,6)
A3		(0.10,1.32,8)	(0.12,0.36,2)	(1,1,1)	(0.12,0.17,0.25)
A4		(0.12,0.46,6)	(0.16,0.58,2)	(4,5.59,8)	(1,1,1)

**Table 18:** Fuzzy numbers of the alternative locations ratings related to criterion C7

	A1	A2	A3	A4
A1	(1,1,1)	(1,3.27,8)	(2,5.73,10)	(1,2.46,6)
A2	(0.12,0.30,2)	(1,1,1)	(0.25,1.70,6)	(0.12,0.58,8)
A3	(0.10,0.17,0.50)	(0.16,0.58,4)	(1,1,1)	(0.25,0.82,6)
A4	(0.16,0.40,2)	(0.12,1.70,8)	(0.16,1.21,4)	(1,1,1)

**Table 19:** Fuzzy numbers of the alternative locations ratings related to criterion C8

	A1	A2	A3	A4
A1	(1,1,1)	(2,4.71,8)	(1,2.08,4)	(0.12,0.52,2)
A2	(1,1.91,8)	(1,1,1)	(0.12,0.36,2)	(1,2.46,6)
A3	(0.25,0.48,2)	(0.16,0.28,0.5)	(1,1,1)	(1,2.76,8)
A4	(0.12,0.21,5)	(0.16,0.4,2)	(2,3.55,6)	(1,1,1)

The values of fuzzy synthetic extent of eight criteria with respect to the goal are calculated below by using Eqs. (8) and (9).

$${\text{Sc}}1 \, = \, \left( {10.582; \, 18.48; \, 45} \right) \times \, \left( {1/336;1/92.32;1/34.36} \right) = \, \left( {0.031; \, 0.2; \, 1.31} \right)$$

$${\text{Sc}}2 \, = \, \left( {5.08; \, 15.89; \, 43} \right) \times \, \left( {1/336;1/92.32;1/34.36} \right) = \, \left( {0.015; \, 0.172; \, 1.251} \right)$$

$${\text{Sc}}3 \, = \, \left( {2.09; \, 8.19; \, 41} \right) \times \, \left( {1/336;1/92.32;1/34.36} \right) = \, \left( {0.006; \, 0.089; \, 1.193} \right)$$

$${\text{Sc}}4 \, = \, \left( {2; \, 6.98; \, 38} \right) \times \, \left( {1/336;1/92.32;1/34.36} \right) = \, \left( {0.006; \, 0.076; \, 1.106} \right)$$

$${\text{Sc}}5 \, = \, \left( {4.76; \, 13.36; \, 47} \right) \times \, \left( {1/336;1/92.32;1/34.36} \right) = \, \left( {0.014; \, 0.145; \, 1.368} \right)$$

$${\text{Sc}}6 \, = \, \left( {3.90; \, 9.30; \, 29.16} \right) \times \, \left( {1/336;1/92.32;1/34.36} \right) = \, \left( {0.012; \, 0.101; \, 0.849} \right)$$

$${\text{Sc}}7 \, = \, \left( {3.84; \, 11.83; \, 43.5} \right) \times \, \left( {1/336;1/92.32;1/34.36} \right) = \, \left( {0.011; \, 0.128; \, 1.266} \right)$$

$${\text{Sc}}8 \, = \, \left( {2.07; \, 8.28; \, 49.5} \right) \times \, \left( {1/336;1/92.32;1/34.36} \right) = \, \left( {0.006; \, 0.09; \, 1.441} \right)$$

The *V* values for the C_1_ calculated using Eq. (13).

$${\text{V}}\left( {{\text{S}}_{{{\text{c}}1}} > {\text{ S}}_{\text{c2}} } \right) = \, 1$$

$${\text{V}}\left( {{\text{S}}_{{{\text{c}}1}} > {\text{ S}}_{{{\text{c}}3}} } \right) = \, 1$$

$${\text{V}}\left( {{\text{S}}_{{{\text{c}}1}} > {\text{ S}}_{{{\text{c}}4}} } \right) = \, 1$$

$${\text{V}}\left( {{\text{S}}_{{{\text{c}}1}} > {\text{ S}}_{{{\text{c}}5}} } \right) = \, 1$$

$${\text{V}}\left( {{\text{S}}_{{{\text{c}}1}} > {\text{ S}}_{{{\text{c}}6}} } \right) = \, 1$$

$${\text{V}}\left( {{\text{S}}_{{{\text{c}}1}} > {\text{ S}}_{{{\text{c}}7}} } \right) = \, 1$$

$${\text{V}}\left( {{\text{S}}_{{{\text{c}}1}} > {\text{ S}}_{{{\text{c}}8}} } \right) = \, 1$$

For the rest of the values calculations are presented in Appendix (A).

The minimum degree of possibility of superiority of each criterion over another is obtained by using Eq. (14).

$${\text{m}}({\text{C}}_{1} ) \, = \, \hbox{min} V({\text{S}}_{\text{i}} \ge {\text{S}}_{\text{k}} ) = \hbox{min} \left( {1;\;1;\;1;\;1;\;1;\;1;\;1;\;1} \right) = 1$$

Similarly; m(C_2_) = 0.978, m(C_3_) = 0.912, m(C_4_) = 0.896,

$${\text{m}}({\text{C}}_{5} ) = \, 0.960, \quad {\text{ m}}({\text{C}}_{6} ) = \, 0.892, \quad {\text{ m}}({\text{C}}_{7} ) = \, 0.945\quad {\text{ and m}}({\text{C}}_{8} ) = \, 0.927$$

Therefore, the weight vector is given as: W′ = (1; 0.978; 0.912; 0.896; 0.960; 0.892; 0.945; 0.927).

The normalized weight vectors are calculated as: W = (0.133, 0.13, 0.122, 0.128, 0.128, 0.119, 0.126, 0.123).

Calculation of the weight vectors for the alternative evaluation matrices followed the same calculation. The normalized weight vectors of alternative locations from Tables 15–19 and weight of criteria are summarized in Table 20. For location alternative A1, the global performance was computed as:

$$\begin{aligned} {\text{D}}\left({{\text{A}}_{1}} \right) = ({\text{d}}\left({{\text{A}}_{{1{\text{C}}1}}} \right) \times {\text{d}}\left({{\text{C}}_{1}} \right) + {\text{d}}({\text{A}}_{{1{\text{C}}2)}} \times {\text{d}}\left({{\text{C}}_{2}} \right) + {\text{d}}\left({{\text{A}}_{{1{\text{C}}3}}} \right) \times {\text{d}}\left({{\text{C}}_{3}} \right) + {\text{d}}\left({{\text{A}}_{{1{\text{C}}4}}} \right) \times {\text{d}}\left({{\text{C}}_{4}} \right) + {\text{d}}\left({{\text{A}}_{{1{\text{C}}5}}} \right) \times {\text{d}}\left({{\text{C}}_{5}} \right) {\text{d}}\left({{\text{A}}_{{1{\text{C}}6}}} \right) \times {\text{d}}\left({{\text{C}}_{6}} \right) \hfill \\ + {\text{d}}\left({{\text{A}}_{{1{\text{C}}7}}} \right) \times {\text{d}}\left({{\text{C}}_{7}} \right) + {\text{d}}\left({{\text{A}}_{{1{\text{C}}8}}} \right) \times {\text{d}}\left({{\text{C}}_{8}} \right) = 0.3004 \hfill \\ \end{aligned}$$

**Table 20:** Weight vectors of the criteria and alternative locations

	C1	C2	C3	C4	C5	C6	C7	C8
A1	0.260	0.286	0.330	0.405	0.270	0.253	0.298	0.279
A2	0.246	0.261	0.270	0.324	0.243	0.261	0.244	0.256
A3	0.249	0.223	0.229	0.143	0.237	0.217	0.217	0.233
A4	0.245	0.229	0.170	0.127	0.250	0.269	0.242	0.232
Weight of criteria	0.133	0.130	0.122	0.128	0.128	0.119	0.126	0.123

The global performance for the other alternative locations was calculated similarly. Table 21 presents the global performance for all the alternatives and their ranking position. Consequently, following this process, similarly to the application of Fuzzy TODIM, location A_1_ is the best evaluated alternative, followed by A_2_, A_3_, and A_4_.

**Table 21:** Global performance of alternative locations and ranking

	Global performance	Rank
A1	0.3004	1
A2	0.2655	2
A3	0.2205	3
A4	0.2224	4

### Comparative analyses

Fuzzy AHP and fuzzy TODIM methods are both appropriate for the selection of a landfill location. However, these two methods have some shortcomings and advantages. According to the nature of this problem, the most appropriate method should be chosen.

We can summarize the differences and similarities between fuzzy AHP and fuzzy TODIM methods as follows:

1. When both these methods of multi-criteria decision-making are compared in relation to the amount of calculations, fuzzy AHP requires more complex calculation than fuzzy TODIM.
2. Pairwise comparison matrices for criteria and alternatives are performed in fuzzy AHP, while there is no pairwise comparison in fuzzy TODIM (Tosun and Akyüz 2014; Salomon and Rangel 2015).
3. The Fuzzy TODIM technique does not require any limit to the number of criteria or alternatives used in the decision making process. On the contrary, the comparative analysis of the Fuzzy AHP technique requires some limitation on the number of criteria and alternatives. Saaty (1980) indicates that the number of decision alternatives or criteria to be compared by using AHP should be limited to 9 so as not to compromise human judgment and its consistency. This proposition also applies to the Fuzzy AHP technique.

In the application case, with eight criteria and four alternatives, the use of the Fuzzy AHP technique was perfectly good. Consequently, the selection of the technique depends on the specificities of the circumstances at hand. For example, when selecting a new location for landfill waste, with many potential alternatives and criteria, the Fuzzy TODIM is a best choice.

4. In the analysis of fuzzy AHP technique (Chang 1992, 1996), the weights of alternative or criterion may be null. In that case, we do not take this criterion or this alternative into account. This is the one of the drawbacks of this technique.
5. The classification results of the fuzzy AHP and fuzzy TODIM are identical. This shows that when the decision-makers are consistent with himself in specifying the data, both techniques independently, the classification results will be identical.
6. We can adopt linguistic variables for the two methods: fuzzy AHP and fuzzy TODIM.
7. Fuzzy TODIM method ranks alternatives measuring their values of gain and loss, providing then a meaningful overall value for each alternative. In fuzzy AHP method, decision-makers elaborate pairwise comparisons and the weights of alternatives are computed by using the Chang’ extent analysis procedure.
8. Both techniques use fuzzy set theory to deal with incertitude and imprecision of the data used in the landfill location selection decision process. In both techniques, the fuzzy number is the main resource for quantifying vagueness. Because of the imprecision of judgments of qualitative variables, the values of the triangular fuzzy number can be selected so as to better represent the linguistic terms used by each expert to assess the alternatives in regard to different decision criteria.
9. Two techniques allow aggregation of judgments by many experts. In the application case of the Fuzzy TODIM technique, aggregation of different judgments is elaborate according to Eqs. (19) and (20) for the weights of the criteria and the ranking of the alternative locations. In the application of the fuzzy AHP, although this is not explicitly considered in the method proposed by Chang (1992, 1996), which propose that aggregation be made using the geometric mean of the judgments. The necessary amount of data needed by fuzzy AHP technique is higher than that needed by fuzzy TODIM. Increasing the number of experts will therefore cause a greater increase in the time complexity of the fuzzy AHP when compared with the fuzzy TODIM technique. The two techniques support decision-making group, because of the impact on time complexity, therefore, fuzzy TODIM technique is preferable.

## Conclusion

By using fuzzy AHP and fuzzy TODIM, uncertainty and imprecision from subjective and the experiences of experts may be effectively represented and reached to a more efficiently decision. The current study presents a methodological overview of the use of multi-criteria decision making using fuzzy AHP and fuzzy TODIM methods for the landfill location selection. By using different techniques and functions, this study enables decision-makers to resolve the problem of landfill location selection in a more objective way. The importance criteria were land cost, available transportation, distance from residential areas, distance from historical areas, ground water quality, soil type, infrastructure cost and distance from wells. These criteria were assessed to specify the ranking of alternative locations for selecting the most suitable one. Although both techniques have the same goal of selecting the best landfill location for the region, they show differences. In fuzzy TODIM technique, experts use the linguistic terms to evaluate the importance of the criteria and to assess each alternative according to each criterion. These linguistic terms are translated into triangular fuzzy numbers and fuzzy decision matrix was made. Then the values of loss and gain for each alternative were calculated. After the dominance degree of gain (Φ+) and the dominance degree of loss (Φ−) were defined, overall dominance degree matrix was computed. Then, the overall value (δ) of each alternative was computed separately. With respect to the overall value of four alternatives, the ranking order of four alternatives has been specified as A_1_ > A_2_ > A_3_ > A_4_. In fuzzy AHP, experts elaborate pairwise comparisons for the criteria and alternatives according to each criterion. Then these integrated comparisons and experts’ pairwise comparison values were translated into triangular fuzzy numbers. The weights of criteria and alternatives are calculated by Chang’s (1992, 1996) extent procedure. With respect to the combination of the priority weights of criteria and alternatives, the optimal alternative is specified. According to the fuzzy AHP, the optimal alternative location is A_1_ and the ranking of the alternatives is A_1_ > A_2_ > A_3_ > A_4_, the same as fuzzy TODIM. The governmental authorities in Casablanca region should choose the suitable technique for their problem according to the situation and the structure of the problem they have.

Finally, some real contributions of this study can be highlighted.

- It is the first study to analyze the reliability of MCDM techniques to the problem of landfill location selection considering the alignment of the characteristics of the problem with the features of the techniques. A study such as this may help the researchers, the stakeholders and the decision-makers to select more models that are effective to landfill location selection.
- It is the first comparative study to review and bring numeric examples of fuzzy TODIM with other multi-criteria methods such as fuzzy AHP. Forthcoming research could test other fuzzy methods such as fuzzy TOPSIS and VIKOR.
- This study includes other comparative criteria such as modeling of uncertainty and adequacy to supporting decision-making groups. It can also be applied to other multi-criteria decision problems such as software selection, supply chain selection and supplier selection.

## Appendix

Appendix (A) The rest of the degrees of possibility for the criteria from the C_2_ to C_8_, computed as in Eqs. (8), (9) are:

**Table:**

V(Sc2 > Sc1) = 0.978	V(Sc3 > Sc1) = 0.912	V(Sc4 > Sc1) = 0.896	V(Sc5 > Sc1) = 0.960
V(Sc2 > Sc3) = 1	V(Sc3 > Sc2) = 0.934	V(Sc4 > Sc2) = 0.919	V(Sc5 > Sc2) = 0.980
V(Sc2 > Sc4) = 1	V(Sc3 > Sc4) = 1	V(Sc4 > Sc3) = 0.988	V(Sc5 > Sc3) = 1
V(Sc2 > Sc5) = 1	V(Sc3 > Sc5) = 0.955	V(Sc4 > Sc5) = 0.940	V(Sc5 > Sc4) = 1
V(Sc2 > Sc6) = 1	V(Sc3 > Sc6) = 0.990	V(Sc4 > Sc6) = 0.978	V(Sc5 > Sc6) = 1
V(Sc2 > Sc7) = 1	V(Sc3 > Sc7) = 0.968	V(Sc4 > Sc7) = 0.954	V(Sc5 > Sc7) = 1
V(Sc2 > Sc8) = 1	V(Sc3 > Sc8) = 0.999	V(Sc4 > Sc8) = 0.987	V(Sc5 > Sc8) = 1
V(Sc6 > Sc1) = 0.892	V(Sc7 > Sc1) = 0.945	V(Sc8 > Sc1) = 0.927	
V(Sc6 > Sc2) = 0.921	V(Sc7 > Sc2) = 0.966	V(Sc8 > Sc2) = 0.945	
V(Sc6 > Sc3) = 1	V(Sc7 > Sc3) = 1	V(Sc8 > Sc3) = 1	
V(Sc6 > Sc4) = 1	V(Sc7 > Sc4) = 1	V(Sc8 > Sc4) = 1	
V(Sc6 > Sc5) = 0.950	V(Sc7 > Sc5) = 0.987	V(Sc8 > Sc5) = 0.963	
V(Sc6 > Sc7) = 0.968	V(Sc7 > Sc6) = 1	V(Sc8 > Sc6) = 0.992	
V(Sc6 > Sc8) = 1	V(Sc7 > Sc8) = 1	V(Sc8 > Sc7) = 0.974	

Therefore, the weight vector W′ is:

$$\begin{aligned} {\text{d}}^{{\prime }} \left( {{\text{C}}1} \right) \, & \, = {\text{ V}}\left( {{\text{Sc}}1 > {\text{Sc}}2,{\text{Sc}}3,{\text{Sc}}4,{\text{Sc}}5,{\text{Sc}}6,{\text{Sc}}7,{\text{Sc}}8} \right) \\ & = { \hbox{min} }\left( {1, \, 1, \, 1, \, 1, \, 1, \, 1, \, 1} \right) \, = 1 \\ \end{aligned}$$

$$\begin{aligned} {\text{d}}^{{\prime }} \left( {{\text{C}}2} \right) \, & = {\text{ V }}\left( {{\text{Sc}}2 > {\text{ Sc}}1,{\text{ Sc}}3,{\text{ Sc}}4,{\text{ Sc}}5,{\text{ Sc}}6,{\text{ Sc}}7,{\text{ Sc}}8} \right) \\ & = { \hbox{min} }\left( {0.978, \, 1, \, 1, \, 1, \, 1, \, 1, \, 1} \right) \, = 0.978 \\ \end{aligned}$$

$$\begin{aligned} {\text{d}}^{{\prime }} \left( {{\text{C}}3} \right) \, & = {\text{ V}}\left( {{\text{Sc}}3 > {\text{ Sc}}1,{\text{ Sc}}2,{\text{ Sc}}4,{\text{ Sc}}5,{\text{ Sc}}6,{\text{ Sc}}7,{\text{ Sc}}8} \right) \\ & = { \hbox{min} }\left( {0.912, \, 0.934, \, 1, \, 0.955, \, 0.990, \, 0.968, \, 0.999} \right) \, = 0.912 \\ \end{aligned}$$

$$\begin{aligned} {\text{d}}^{{\prime }} \left( {{\text{C}}4} \right) \, & = {\text{ V}}\left( {{\text{Sc}}4 > {\text{ Sc}}1,{\text{ Sc}}2,{\text{ Sc}}3,{\text{ Sc}}5,{\text{ Sc}}6,{\text{ Sc}}7,{\text{ Sc}}8} \right) \\ & = { \hbox{min} }\left( {0.896, \, 0.919, \, 0.988, \, 0.940, \, 0.978, \, 0.954, \, 0.987} \right) \, = 0.896 \\ \end{aligned}$$

$$\begin{array}{*{20}l} {{\text{d}}^{\prime}\left({{\text{C}}5} \right) = {\text{V}}\left({{\text{Sc}}5 > {\text{Sc}}1,{\text{Sc}}2,{\text{Sc}}3,{\text{Sc}}4,{\text{Sc}}6,{\text{Sc}}7,{\text{Sc}}8} \right)} \hfill \\ {\qquad \quad = \hbox{min} \left({0.960, 0.980, 1, 1, 1, 1, 1} \right) = 0.960} \hfill \\ \end{array}$$

$$\begin{aligned} \hfill {\text{d}}^{{\prime }} \left( {{\text{C}}6} \right) &= {\text{ V }}\left( {{\text{Sc}}6 > {\text{ Sc}}1,{\text{ Sc}}2,{\text{ Sc}}3,{\text{ Sc}}4,{\text{ Sc}}5,{\text{ Sc}}7,{\text{ Sc}}8} \right) \\ \hfill &= {\hbox{min} }\left( {0.892, \, 0.921, \, 1, \, 1, \, 0.950, \, 0.968, \, 1} \right) \, = 0.892 \\ \end{aligned}$$

$$\begin{aligned} {\text{d}}^{{\prime}} \left({{\text{C}}7} \right)& = {\text{V}}\left({{\text{Sc}}7 > {\text{Sc}}1,{\text{Sc}}2,{\text{Sc}}3,{\text{Sc}}4,{\text{Sc}}5,{\text{Sc}}6,{\text{Sc}}8} \right) \hfill \\ &= { \hbox{min} }\left({0.945, 0.966, 1, 1, 0.987, 1, 1} \right) = 0.945 \hfill \\ \end{aligned}$$

$$\begin{aligned} {\text{d}}^{{\prime }} \left( {{\text{C}}8} \right) & = {\text{ V}}\left( {{\text{Sc}}8 > {\text{ Sc}}1,{\text{ Sc}}1,{\text{ Sc}}3,{\text{ Sc}}4,{\text{ Sc}}5,{\text{ Sc}}6,{\text{ Sc}}7} \right) \hfill \\ &= { \hbox{min} }\left( {0.927, \, 0.945, \, 1, \, 1, \, 0.963, \, 0.992, \, 0.974} \right) \, = 0.927 \hfill \\ \end{aligned}$$

$$W^{{\prime }} \, = \, \left( {1; \, 0.978; \, 0.912; \, 0.896; \, 0.960; \, 0.892; \, 0.945; \, 0.927} \right)$$
